# Low leopard populations in protected areas of Maputaland: a consequence of poaching, habitat condition, abundance of prey, and a top predator

**DOI:** 10.1002/ece3.2771

**Published:** 2017-02-23

**Authors:** Tharmalingam Ramesh, Riddhika Kalle, Havard Rosenlund, Colleen T. Downs

**Affiliations:** ^1^ School of Life SciencesUniversity of KwaZulu‐NatalScottsvillePietermaritzburgKwaZulu‐NatalSouth Africa; ^2^ School of Ecology and Environment StudiesNalanda UniversityRajgirIndia

**Keywords:** Africa, Bayesian approach, competition, poaching, prey abundance, spatially explicit capture–recapture, threats

## Abstract

Identifying the primary causes affecting population densities and distribution of flagship species are necessary in developing sustainable management strategies for large carnivore conservation. We modeled drivers of spatial density of the common leopard (*Panthera pardus*) using a spatially explicit capture–recapture—Bayesian approach to understand their population dynamics in the Maputaland Conservation Unit, South Africa. We camera‐trapped leopards in four protected areas (PAs) of varying sizes and disturbance levels covering 198 camera stations. Ours is the first study to explore the effects of poaching level, abundance of prey species (small, medium, and large), competitors (lion *Panthera leo* and spotted hyenas *Crocuta crocuta*), and habitat on the spatial distribution of common leopard density. Twenty‐six male and 41 female leopards were individually identified and estimated leopard density ranged from 1.6 ± 0.62/100 km^2^ (smallest PA—Ndumo) to 8.4 ± 1.03/100 km^2^ (largest PA—western shores). Although dry forest thickets and plantation habitats largely represented the western shores, the plantation areas had extremely low leopard density compared to native forest. We found that leopard density increased in areas when low poaching levels/no poaching was recorded in dry forest thickets and with high abundance of medium‐sized prey, but decreased with increasing abundance of lion. Because local leopard populations are vulnerable to extinction, particularly in smaller PAs, the long‐term sustainability of leopard populations depend on developing appropriate management strategies that consider a combination of multiple factors to maintain their optimal habitats.

## 1 Introduction

Large wide‐ranging carnivores naturally occur at low densities due to their slow recruitment rates and specialized habitat requirements (Gros, Kelly, & Caro, 1996; Hayward, O'Brien, & Kerley, 2007; Karanth, Nichols, Kumar, Link, & Hines, 2004; Ray, Hunter, & Zigouris, 2005). Habitat loss, fragmentation, and degradation of natural habitat from large‐scale plantations, retaliatory killing, and poaching are some of the major threats to the survival of carnivores and their prey populations (Balme, Slotow, & Hunter, 2010; Lantschner, Rusch, & Hayes, 2012; Ramesh, Kalle, Rosenlund, & Downs, 2016; Swanepoel et al., 2014). Over time, these threats have reduced their densities, increased their dependency on protected areas (PAs), decreased their population viability, and increased their extinction risks (Sankar et al., 2010; Woodroffe & Ginsberg, 1998).

For example, common leopard (*Panthera pardus*) populations are declining, and have disappeared from nearly 40% of their historic African range (Henschel et al., 2008; Ray et al., 2005). Range contraction of leopard is prevalent in South Africa (Swanepoel, Lindsey, Somers, Hoven, & Dalerum, 2013). In critical ecosystems where poaching is prevalent, identifying the primary causes affecting low population densities, distribution, and other causes of mortality are necessary in developing sustainable management strategies for large carnivore conservation. Although carnivore density is positively correlated with prey abundance (Karanth et al., 2004), additive effects of competing top predators may impact populations of other predators through kleptoparasitism, injury, and direct mortality (Caro & Stoner, 2003; Donadio & Buskirk, 2006; Mills & Mills, 1982). Such competition can reduce the population size of an endangered carnivore through risk of dominant competitors that are larger or live in competitively dominant social groups; for instance, lions (*Panthera leo*) and spotted hyenas (*Crocuta crocuta*) have negative impacts on populations of African wild dogs (*Lycaon pictus*), cheetah (*Acinonyx jubatus*) (Creel & Creel, 1996; Durant, 2000), and leopard (Mills, 2015). Furthermore, “fear” instilled in other predators by apex predators can alter the former's habitat use (Brown, Laundre, & Gurung, 1999; Durant, 2000), activity, and dispersal patterns (Donadio & Buskirk, 2006). These interactions can thus become additional determinants of species distribution and abundance and help shape community structure and ecosystem function.

Protected area size plays an important role in shaping wildlife populations due to the edge effects of human disturbances surrounding PAs. This is evident through high mortality of large carnivores even inside PAs, which affect resident carnivores and prey species (Balme et al., 2010; Sankar et al., 2010; Woodroffe & Ginsberg, 1998). Carnivore densities can therefore vary in an ecosystem in relation to various aspects of prey, co‐predators, and disturbance levels. Spatially explicit capture–recapture (SECR) models are increasingly advancing the field of population ecology (Efford, 2004; Royle, Karanth, Gopalaswamy, & Kumar, 2009; Royle & Young, 2008) and are less biased than conventional closed capture–recapture methods by study design, sample sizes, and variation in detection probabilities for effective conservation and management (Ramesh & Downs, 2013; Sollmann, Gardner, & Belant, 2012). SECR models generate convincing inferences with low sample sizes under the Bayesian framework (Alexander, Gopalaswamy, Shi, & Riordan, 2015; Gopalaswamy et al., 2012) which have been applied to account for the external variable effects on the density of other carnivores, such as black bear (*Ursus americanus*) (Howe, Obbard, & Kyle, 2013), Amur leopard (*Panthera pardus orientalis*) (Qi et al., 2015), and snow leopard (*Panthera uncia*) (Alexander et al., 2015).

Protected areas in Maputaland, South Africa, are important for conservation of leopard as they support one of the few remaining large leopard populations, despite rising anthropogenic pressures in South Africa (Balme, Hunter, & Slotow, 2009; Swanepoel et al., 2013). Although a few studies in Africa have applied SECR models to leopard density estimates (Chase Grey, Kent, & Hill, 2013; Swanepoel, Somers, & Dalerum, 2015), none have accounted for variable effects such as prey abundance, food distribution, co‐occurring species, human disturbance, topography, bioclimate, and other associated threats/factors of interest to quantify spatial distributions of density. We modeled the spatial ecological drivers of leopard density using a Bayesian‐SECR approach to understand the population dynamics of leopards in PAs in Maputaland. We camera‐trapped leopard using a framework of spatial capture–recapture surveys across PAs in the Maputaland Conservation Unit (MCU) to provide reliable leopard density estimates. Firstly, we estimated the densities of leopard populations using Bayesian‐SECR models. Then, we assessed these spatial relationships in the context of poaching pressure, competitor abundance, prey abundance, and habitat types. We predicted that leopard density and its spatial distribution would decrease with increasing poaching pressure and competitor abundance (i.e., lion and hyena detection rate) and positively affected by the relative abundance of prey species (i.e., prey detection rate). We predicted that differences in vegetation characteristics (habitat types) are the main factors contributing to the spatial distribution of leopard density and expected lower densities of leopard in plantation versus native forests in the PAs of the MCU. Our study resulted in significant conservation implications for understanding those multiscale factors affecting carnivore populations, which are applicable worldwide.

## 2 Methods

### 2.1 Study area

**Figure 1:**
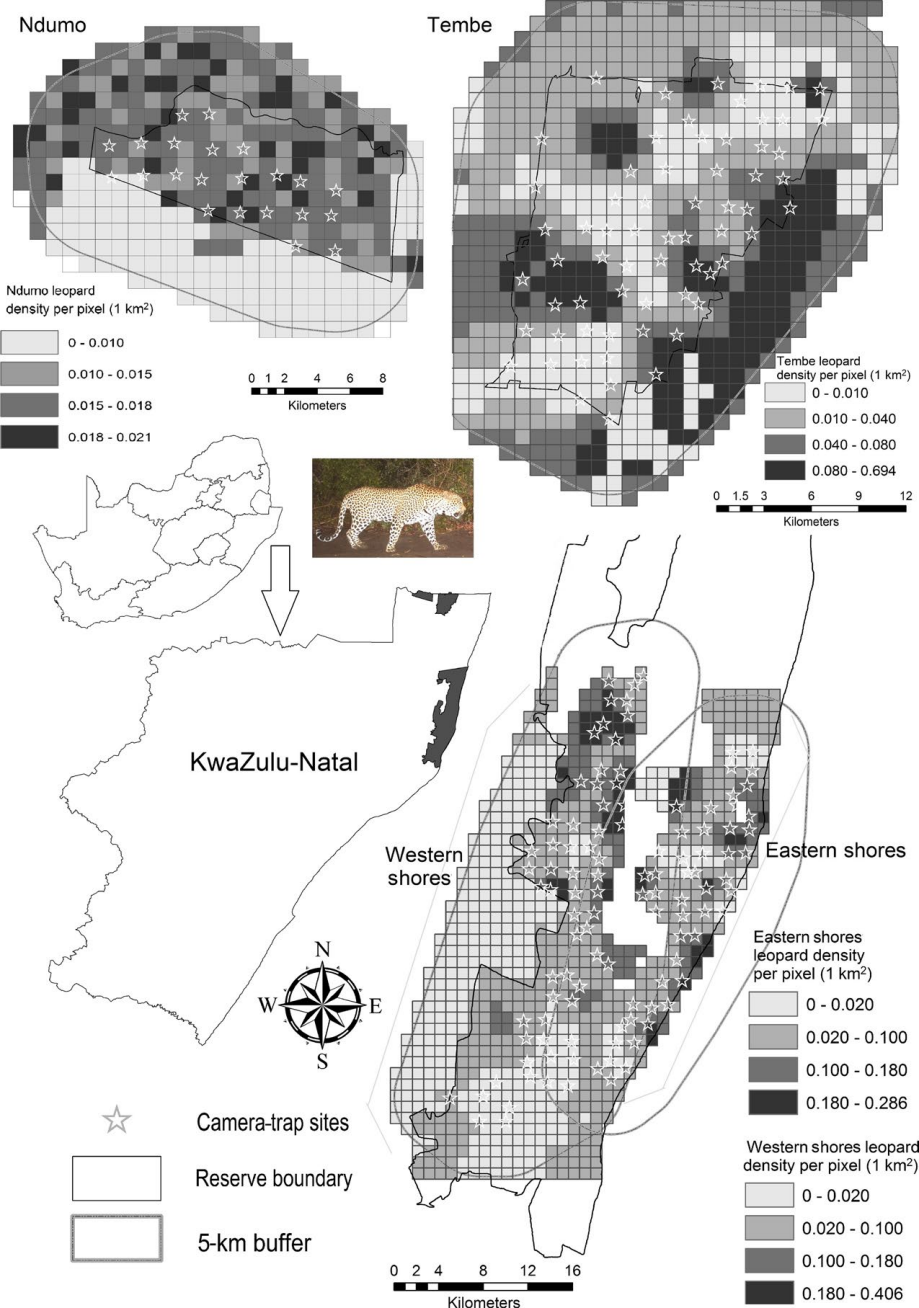
Estimated pixel level density map of the spatial distribution of leopards across the study area in the Maputaland Conservation Unit of South Africa

Our camera‐trap surveys were conducted in five PAs in the MCU in the northern part of KwaZulu‐Natal (KZN) Province, South Africa, covering a total of 198 camera stations between July and April during 2013–2014 (Table S1; Figure 1). Maputaland is an important biodiversity hot spot because of its high endemism (Jones, 2006; Matthews, van Wyk, van Rooyen, & Botha, 2001). Each survey in a PA was conducted for 24–46 days using multiple camera‐trap stations, the number of which varied relative to PA size (Table S1). We completed two surveys in different portions of St. Lucia Wetland Park, a part of iSimangaliso Wetland Park World Heritage Site, including eastern shores (ca. 30,000 ha) and western shores (ca. 38,000 ha) (hereafter, each of these survey areas is considered as “PA”). Of these, western shores is most impacted by human disturbance, as much natural habitat has been destroyed by large‐scale plantation activities (mainly *Eucalyptus* spp.). We conducted our third survey in Tembe Elephant Reserve (Tembe) (ca. 30,000 ha), which is situated in the Maputaland coastal plain midway from the sea to the east, and the Lebombo mountain range to the west. Our fourth survey occurred in Ndumo Game Reserve (Ndumo) (ca. 10,117 ha), which borders Mozambique to the north along the Usutu River, and lies close to Swaziland to the west. Except for the eastern shores, all other areas have human settlements, cultivated lands, and cattle farms on the boundaries of the reserves; Ndumo in particular has the highest poaching pressure and number of human settlements (Jones, 2006).

### 2.2 Data collection and analyses

We deployed passive infrared camera traps (LtI Acorn model 6210MC, China; and Moultrie model M880, USA) to record mammals occurring at all study sites. We considered our sampling area coverage adequate enough to capture multiple leopard home ranges as it was several times larger than the average female home range size (15 km^2^) (Bailey, 1993; Chase‐Grey, 2011). Moreover, we ensured that at least four to six camera‐trap sites operated in each home range to maximize the chance of capturing different individuals found within the sampling area, and to ensure capture independence among neighboring trap stations. Camera‐trap surveys were conducted in all sites using a systematic grid (2 km^2^) covering various habitat types and disturbed areas, with an average intertrap distance of 1.5 km to maximize the recapture of different individuals at each station (Kalle, Ramesh, Qureshi, & Sankar, 2011; Ramesh, Kalle, Sankar, & Qureshi, 2012b). To select the final camera station positions, we conducted ground surveys to identify locations that fell in each grid and maximize the possibility of detecting leopards based on indirect signs. Camera‐trap stations were positioned facing an active wildlife trail and then secured to a tree at a height of 20 cm from above the ground and about 2–3 m away from the trail. Cameras were left to operate for 24 h daily along roads and animal path/trail for 24–46 days (<2 months/study site) and placed them at a height suitable to photograph species ranging from rodents to elephants, permitting us to quantify prey abundance from camera‐trap data. We distributed the trap stations uniformly across sample sites with varying habitat gradients and performed individual identification of leopards photographed taking the flank region.

We used a Bayesian‐SECR logistic regression model for binary observations and encounter histories for individual leopards captured at a given camera‐trap station during sampling occasions (Gopalaswamy et al., 2012). We estimated leopard densities following Royle et al. (2009) using the package SPACECAP (Gopalaswamy et al., 2014) in program R (R Core Team 2014). Individual leopards were distinguished using natural markings on the flanks, legs, face, and tail (Kalle et al., 2011; Ramesh et al., 2012b). Dates and times for individual marked leopards were recorded during first capture and on subsequent recapture and sexed and aged (Balme, Hunter, & Braczkowski, 2012). Capture histories were created for individuals and formatted for analysis following Gopalaswamy et al. (2012).

We generated state‐space home range center to calculate effective camera‐trapping areas of each sampled survey region, including a 5‐km buffer distance around the sampled area, which was an area larger than the mean maximum distance moved (MMDM) by recaptured individuals between camera traps. Buffer size was based on the MMDM by individuals from camera traps and was sufficient to contain several individual home ranges (Kalle et al., 2011). Part of the western shores buffer area extended east into eastern shores, and the eastern shores region buffer area extended west into western shores through Lake St. Lucia, separating the connection between both. We used data from eastern shores and western shores separately to predict leopard density (see Ramesh et al., 2016; for study area view). The state space was described using equally spaced points in a regular grid and a mesh size of 1 km^2^. We identified nonhabitat area within a particular grid using land cover overlaid on the study area, marked as “0” in the input file. We also considered unsuitable habitat as areas outside the PA, which had a high proportion of human settlements, agricultural land, roads, or development. We ran program SPACECAP using a half‐normal distribution model fitted to the distance between home range centers and trap location: Data augmentation was increased to five times higher than individuals captured for 60,000 iterations a burn‐in of 20,000 and thinning rate of 10. To calculate our Bayesian spatial estimate, we used the Bernoulli distribution, with trap response absent from the analysis (Royle et al., 2009) and assessed model adequacy using a Bayesian *p*‐value of 0 or 1 (Gopalaswamy et al., 2012). The population estimate from the Bayesian‐SECR models is least biased to geographic closure assumption as this model addresses edge effects (odd shapes and sizes) from the sampling areas (Gardner, Royle, Wegan, Rainbolt, & Curtis, 2010; Gopalaswamy et al., 2012; Sollmann et al., 2013). As Bayesian‐SECR models directly estimate animal density by explicitly using the information on capture histories in combination with spatial locations of captures under a unified Bayesian modeling framework (Gopalaswamy et al., 2012), we assumed demographic closure, and thus, we limited our sampling period to <3 months in each study site (Kalle et al., 2011; Ramesh et al., 2012b).

We modeled density estimates with potential site covariates quantified in our survey sites, and reclassified the land cover map (Matthews et al., 2001; iSimangaliso Wetland Park Authority 2014) to include eight broad habitat types: coastal lowland, dry forest thicket, Makatini clay thicket, dune, grassland, plantation, sand forest, and woodland (Ramesh et al., 2016). We used continuous site covariates such as presence of prey (small, medium, and large), competitor (lion and hyena) detection rate, and categorical site covariates, including poaching level (high and low), path (wider/narrow), and habitat (one of the eight), all assumed to influence habitat use of leopards (Ramesh et al., 2016). We extracted habitat variables for each camera station point representative of the immediate area using a 15‐m buffer created around the camera using Zonal Statistics tools in ArcGIS 9.3 (ESRI, Redlands, CA, USA); we also assessed habitat type during fieldwork. We considered photographic rates as a surrogate for prey availability and for levels of interspecific competition between predators at the camera sites. Although African wild dogs compete with leopards occasionally (Creel, Spong, & Creel, 2001), they occurred at very low densities in the study area and were therefore excluded from analysis. We considered “species detection rate” as an index of species abundance where multiple detections of each prey category and co‐top predator (lion and hyena) in a sampling day were considered to be a single detection on the same day per camera site. To achieve the species detection rate, we divided the total number of detections for each species by the total number of trapping days. We classified important principle prey species of leopard into small (5–20 kg), medium (21–50 kg), and large (>50–200 kg) size, based on body mass following Clements, Tambling, Hayward, and Kerley (2014) and Hayward et al. (2006); more details about prey species photo‐trapped in the study area are presented in Ramesh et al. (2016). As poaching could affect the viability of leopard population in Maputaland (Balme et al., 2010), we scored the poaching levels as high or low at every camera station based on field knowledge, and our interactions with experienced field rangers and park mangers feedback during data collection. Experienced field rangers accompanied us to every camera station during data collection, which aided in ranking the poaching level. Despite some constrains in the quantification of poaching because of its sensitivity to park management; we estimated intensity based on the number of incidents of removal of snares set for capturing wild animals, including occurrence of leopard poaching and bush meat hunting. We ranked poaching levels as high and low at every camera station if the number of poaching incidences of evidences occurred frequently (recorded more than once) and rarely occurred or did not occur at all, respectively. As detection probability of leopards can vary across narrow or wider paths in response to use by other large predators (Ramesh et al., 2016), we considered a path “wider” if cameras were placed on dirt roads and park management roads, and “narrow” if cameras were placed on animal paths/trails. Correlations among independent variables were tested using the Pearson correlation coefficient test to avoid multicollinearity. We retained all independent variables after setting a threshold cutoff 0.60 and selected one of two variables which presumed to have higher influence on leopard spatial density based on our field knowledge.

We explored the effect of selected site covariates on leopard spatial density from available covariate information for 198 cells, where we placed cameras in the study area. As the program SPACECAP (Gopalaswamy et al., 2014) generated mean leopard pixel‐specific densities from all iterations in the MCMC analysis, we modeled the influence of covariates on pixel‐specific leopard densities using associated pixel‐specific covariates. We first used a generalized linear model (GLM) with the Poisson distribution family to model the covariate influence on these pixel‐specific leopard densities as this was a continuous response variable. As we observed an overdispersion in our data set using the Poisson family, we then used the negative binomial (θ = 1) family (Hilbe, 2011) to further test the variables influencing leopard abundance and distribution. We generated the best‐fit candidate models with few predictors following the framework of Burnham and Anderson (2002). We identified the best models explaining leopard abundance using the Akaike's information criterion for small sample sizes (AICc), AIC differences, and Akaike weights (w_i_) (Burnham & Anderson, 2002). All candidate models ranking ≤2ΔAIC were selected as best‐fit models for explaining variable influences on leopard abundance (Burnham & Anderson, 2002). The relative influence of each response variable (AICc weight) on leopard abundance varied from 0 (no support) to 1 (complete support) relative to the overall models. These statistical analyses were performed in Program R version 3.0 (R Development Core Team, 2014) using packages MASS (Venables & Ripley, 2002), rJava (Urbanek, 2010), glmulti (Calcagno & de Mazancourt, 2010), MuMIn (Barto′n, 2013), and effects (Fox et al., 2015).

## 3 Results

**Figure 2:**
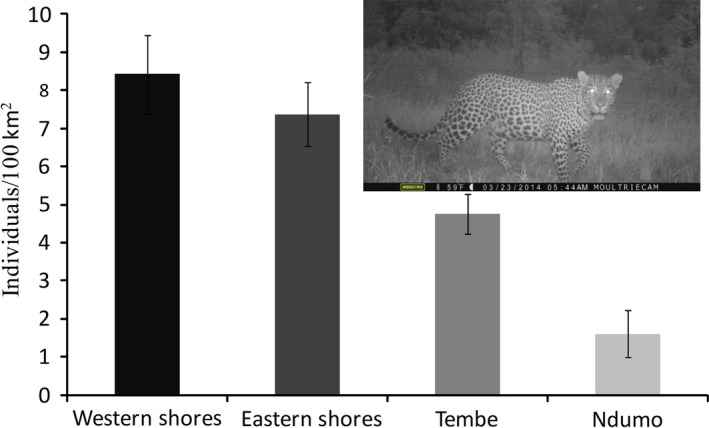
Estimated leopard density per 100 km^2^ across the study area in the Maputaland Conservation Unit of South Africa. Although St. Lucia represents western shore and eastern shore management units, we considered these as different study regions because of the distinct habitat conditions and management systems

**Figure 3:**
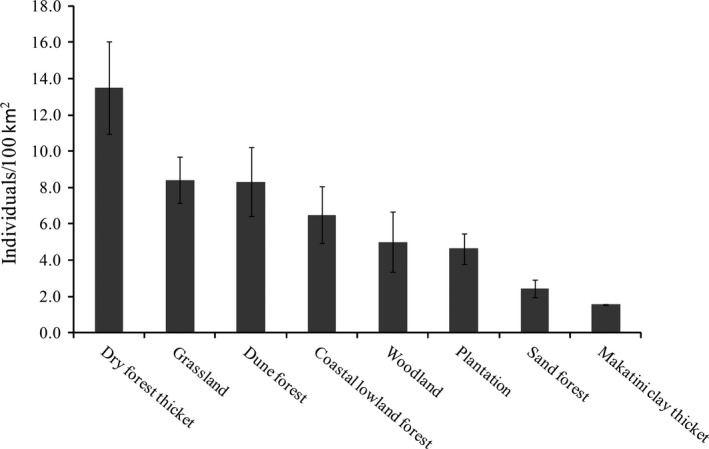
Estimated leopard density per 100 km^2^ across the habitats in the Maputaland Conservation Unit of South Africa

We recorded 266 identifiable and 16 nonidentifiable photographs of leopard captures across all survey regions from 6,209 trap days (Table S1). Sixty‐seven individual leopards were identified, and capture histories were constructed. Number of males and females captured were 26 and 41, respectively. All adult leopard captures were used to calculate leopard densities (age >1 year). Two cubs were recorded along with an adult female and two subadults recorded together on an occasion. Single adult leopards were mostly photographed; however, five photographs had two individuals; male with female and two adult females were captured on different occasions. The MMDM from the recaptured individuals (capture >1) was 3.5 ± 1.14 km and increased in low leopard density areas compared with high‐density areas (Table S1). According to the PA size, the effective sampling area size varied from 70 km^2^ to 312 km^2^ derived from 100 percent minimum convex polygon (MCP 100%) by connecting the boundary camera locations. The summaries of posterior Bayesian‐SECR model estimates fitted with half‐normal detection function are given in Table S2. The mean leopard density for the identified suitable habitat within the 5‐km buffer zone ranged from 1.6/100 km^2^ (with a 95% interval of 1.26–2.93) to 8.4/100 km^2^ (with a 95% interval of 6.77–10.42) for Ndumo and the western shores, respectively (Table S2; Figures 2 and 3). Spatially explicit leopard density distribution maps were created as a raster image with a resolution of 1 km^2^ grid cell or pixel (Figure 1). The pixel level density estimates showed that some areas had several folds higher density of leopard than lower density areas. We found high spatial variation and low leopard density estimates in Ndumo, where poaching level was high throughout. Bayesian *p* values based on individual encounters across PAs confirmed that our models had adequate fit (Tembe = 0.69; Ndumo = 0.53; eastern shores = 0.52; western shores = 0.66). Across the habitats, leopard density was found to be higher in dry forest thickets and lower in plantation, sand forest, and Makatini clay thicket (Figure 3). Although both dry forest thickets and plantation habitats were mainly in the western shores (see Ramesh et al., 2016), plantations had exceptionally low leopard density in comparison with native habitats (Figures 2 and 3).

**Figure 4:**
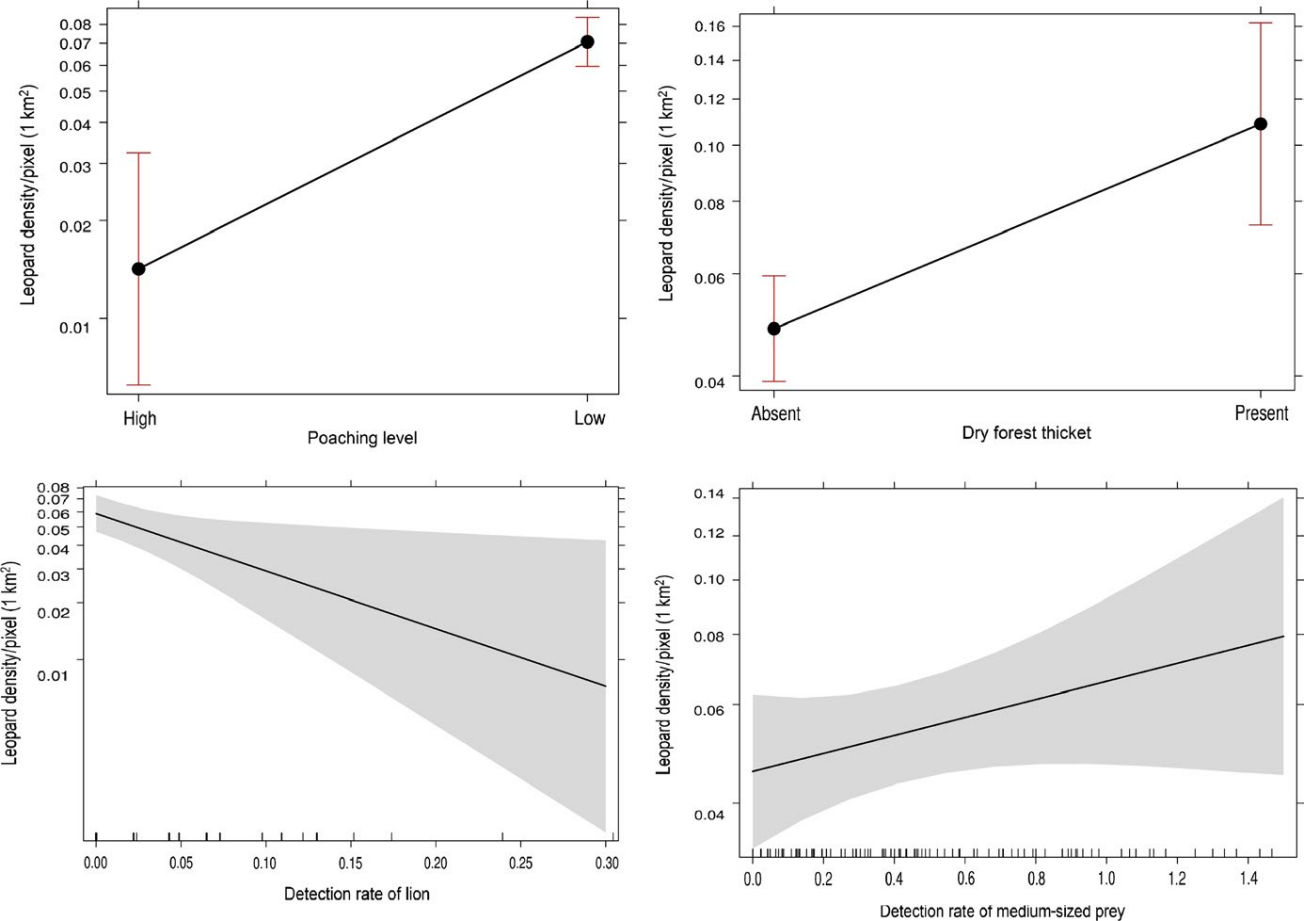
Top generalized linear model with negative binomial family showing the relationship between leopard density (individuals per 1 km^2^) and important predictors

Across all the negative binomial models, poaching (AIC weights = 0.91) was the most influential covariate determining the probability of leopard density within the PAs, followed by dry forest thickets (AIC weights = 0.40), lion (AIC weights = 0.37), medium‐sized prey (AIC weights = 0.22), and hyena (AIC weights = 0.20) (Table S3). Four best candidate models were identified for determining the probability of leopard density based on the models with low AICc (≤2∆AIC). Poaching (estimated coefficient = −1.605, *SE* = 0.426, χ^2^ = 3.77, *p *=* *.000) was identified as one of the strong predictors of leopard density across the four competing models; leopard density increased significantly with low poaching levels or no poaching reported (Figure 4). Leopard densities increased in dry forest thickets but decreased with increasing lion abundance as represented in the top models, with significant relationships (Figure 4; Table S3 and Table 1). In addition, leopard density increased with increasing medium‐sized prey abundance, although the latter had no significant independent influence on leopard density (Figure 4).

**Table 1:** Top generalized linear models (delta‐AIC < 2) with negative binomial family measuring the influence of covariates on estimates of leopard abundance

Model	Parameter	Coefficient	*SE*	*t* value	*p* Value
Leopard density ~Poaching	Intercept	−4.254	0.417	−10.210	<.0001
	Low Poaching	1.605	0.426	3.771	.0002
Leopard density ~DFT + Poaching	Intercept	−4.290	0.407	−10.531	<.0001
	DFT	0.812	0.214	3.789	.0002
	Low Poaching	1.519	0.417	3.643	.0003
Leopard density ~Lion + Poaching	Intercept	−4.230	0.436	−9.693	<.0001
	Lion	−6.981	3.0668	−2.276	.0239
	Low Poaching	1.686	0.450	3.748	.0002
Leopard density ~Medium prey + Poaching	(Intercept)	−4.527	0.4707	−9.617	<.0001
	Medium prey	0.371	0.2622	1.414	.1591
	Low Poaching	1.735	0.4435	3.912	.00013

DFT, Dry forest thicket.

## 4 Discussion

Our systematic camera‐trap surveys and analyses using the robust Bayesian‐SECR approach under the mark–recapture framework estimated the density of common leopard populations in PAs of MCU. Leopard populations ranged from low to high density within MCU. Ours is the first and the most extensive study to estimate the density of common leopard in South Africa, following the Bayesian‐SECR approach as a function of camera site‐specific covariates. Collecting data from several study sites in the same landscape allowed us to assess variation in density among sites and statistically test the causes of variation. The SECR estimate provides the least biased population size when compared with nonspatial methods as the former is flexible with geographic closure assumption and addresses edge effects from the sampling areas (Gardner et al., 2010; Sollmann et al., 2013). Our estimates were reasonable and within the bounds of leopard density generated in South Africa with other approaches.

The low leopard population in Ndumo is vulnerable to local extinction in the near future, due to extensive poaching pressure from adjacent nonprotected areas (Jones, 2006). Ndumo, a small isolated PA surrounded by degraded land, agricultural activity, and cattle ranches, supported low densities of leopard. Leopard population decreased in areas exposed to high poaching pressure across the survey regions. Therefore, leopards seem to be avoiding high‐risk areas or they could be dispersing outside highly disturbed PAs due to the valuable food resources outside the park, despite the considerable foraging cost (Balme et al., 2010). Maputaland is one of the most underdeveloped areas in South Africa, characterized by high poverty levels, low employment status, and limited infrastructure (DEAT 2006). In these PAs, bush meat poaching has increased substantially in recent years due to the lack of inexpensive protein substitutes, trade, and demands from neighboring countries and international market (Jones, 2006). However, we highlight that this negative impact is possibly also mediated through the loss of small livestock from the surrounding community areas, leading to direct persecution of carnivores in this landscape (Meer, 2010). Small livestock farms with high human pressure provide less suitable leopard habitat in South Africa (Swanepoel et al., 2013). Similarly tigers (*Panthera tigris*) in Sariska Reserve, Western India, were exposed to high poaching rates and an absence of immigration resulted in their localized extinction (Sankar et al., 2010). Many monitored leopards in Phinda Reserve, Maputaland, had home ranges outside the reserve and their vulnerability to human persecution on adjacent nonprotected land reduced their probability of survival (Balme et al., 2010). These anthropogenic lethal effects must be accounted when estimating density and distribution of large carnivores throughout their range. As unprotected private land is vital to leopard conservation in Africa, carnivore conservation could be enhanced by increasing the locals’ tolerance to carnivores through education, improving financial aids through ecotourism and setting appropriate management plans to reduce livestock loss.

Through interspecific interactions, lions can reduce the abundance of leopards and alternately utilize shared resources or prey species. As a consequence, social subordinates (in this case leopards) avoid high risk of encounter or predation threat, by using areas where the competing predator densities are lower (Mills, 2015; Mills & Gorman, 1997). Where lion distribution spatially overlapped with leopard, the density of leopard decreased drastically. However, leopards can coexist with sympatric dominant competitors by shifting their temporal activity patterns, prey choice, and habitat selection (Bailey, 1993; Ramesh, Kalle, Sankar, & Qureshi, 2012c), which is yet another dimension of the data to be explored. Cheetah occurs in low densities where their main competitors (lions and spotted hyenas) exist in high densities (Durant, 2000). In South Africa, the park management could hamper the population status of cheetah, leopard, and wild dog as many reintroduction programs are primarily focused on the conservation of lion. Since 2002, lions were reintroduced into Tembe to boost the ecotourism sector. We stress that lion reintroductions could have serious cascading consequences on the abundance of leopard population in Tembe as leopards have been sporadically killed by lions (W. Clinton personal communication). Therefore, the intactness and stability of carnivore populations during reintroductions can potentially elevate the levels of intraspecific competition. Further camera‐trap surveys must be conducted in other parts of Africa to fully understand such top‐down processes.

South Africa has nearly 20% of its habitat suitable for leopard, but these habitats are severely fragmented (Swanepoel et al., 2013). Areas protected according to the International Union for Conservation of Nature (IUCN) criteria comprise only 12% of suitable leopard habitat (Dudley, 2008; Swanepoel et al., 2013). Our findings showed that the distribution of leopard densities varied with habitat types and medium‐sized prey abundance within PAs. The high density of leopards in dry forest thickets of the western shores (13.5/100 km^2^) was comparable to other habitats. Sand forest and Makatini clay thicket had the lowest densities of leopard likely reflecting the higher abundance of lion and increased anthropogenic activities, particularly poaching (Ramesh et al., 2016). The leopard density in plantations of western shores was relatively lower compared with sites having continuous native vegetation. This may be attributed to the reduced understory cover and vegetation diversity and lower availability of refuge and prey within dense plantations of *Eucalyptus spp* (Ramesh et al., 2016). High levels of daily human activities such as burning of vegetation, continuous noise and disturbance from logging trucks, and activity of plantation workers during the day (Ramesh et al., 2016) are additional drivers of low abundance of leopards. Consequently, plantations may not offer the optimum habitat for leopard, which highlights the importance of retaining native vegetation in MCU.

Medium‐sized ungulates constitute the largest proportion of the leopard's diet (Ramesh, Kalle, Sankar, & Qureshi, 2012a). Our analysis showed that the abundance of medium‐sized prey species predicted high leopard density zones in the PAs. The higher occupancy of bushbuck (*Tragelaphus scriptus*), a medium‐sized prey species, in the western shores and eastern shores, was comparable to other PAs (Ramesh et al., 2016), and its solitary nature makes it more vulnerable to predation by leopard (Bailey, 1993; Balme, Hunter, & Slotow, 2007). Other highly abundant medium antelopes such as nyala (*Tragelaphus angasii*) in the dry forest thicket would have supported high leopard density. In Tembe, leopard mainly fed ≥70% of medium‐sized prey that included nyala, impala (*Aepyceros melampus*), and common reedbuck (*Redunca arundinum*) (Wright, 2015). This suggests that the conservation of medium‐sized ungulate prey species is important to enable leopard populations to viably persist.

### 4.1 Conservation implications

Our study demonstrates systematic field sampling surveys to effectively address the impact of associated threats on the spatial distribution of leopard density, using the Bayesian‐SECR approach. We showed the combination of factors such as poaching, habitat type, competitor, and medium‐sized prey, driving the spatial distribution and densities of leopards in MCU. This highlighted the need to develop further rigorous approaches to improve the measurement of leopard density across its distributional range. Priority should be given to improve the viability of smaller reserves to maximize reserve size, corridor linkage, and develop mitigation strategies to reduce carnivore persecution on reserve borders or buffer zones. This conservation effort could be achieved by increasing tolerance levels through educational awareness programs, improving local livelihood conditions and improving financial benefits by promoting tourism, increasing employment, community conservation activities, and mitigation strategies to minimize livestock predation. Our study has applications for habitat prioritization and recovery of native habitat. Other than prey abundance, several other covariates must be addressed in future density estimates of leopard and other carnivore species, for better park management. Although our surveys were limited to the MCU, it still provides managers with current spatial distribution maps of leopard density which has wider applications for carnivore management by the park authorities.

## Conflict of Interest

None declared.
