# Supplementary material for: Low leopard populations in protected areas of Maputaland: a consequence of poaching, habitat condition, abundance of prey, and a top predator
Source: Ecol Evol. 2017 Feb 23;7(6):1964–73. doi: 10.1002/ece3.2771 (PMC5355197; doi:10.1002/ece3.2771)
Supplement: Supplementary file 1 [file ECE3-7-1964-s001.docx]

**Supporting Information**

**Table S1.** Camera trap data collected for leopard population survey from four protected areas in the Maputaland Conservation Unit of South Africa

| Survey details | Western Shore | Eastern Shore | Tembe | Ndumo |
| --- | --- | --- | --- | --- |
| Sampling Area (Km^2^) | 312 | 170 | 294.01 | 70 |
| Trap nights | 1176 | 1488 | 2885 | 660 |
| Average inter-trap distance | 1515 | 1437 | 1803 | 1756 |
| Number of  Individuals captured | 23 | 16 | 25 | 3 |
| Males | 9 | 6 | 10 | 1 |
| Females | 14 | 10 | 15 | 2 |
| Independent detections | 67 | 55 | 136 | 10 |
| Mean maximum distance moved | 2.63±0.808 | 2.96±1.26 | 4.12±1.14 | 4.52±1.47 |

**Table S2.** Posterior summaries of the parameters from Bayesian spatially explicit capture-recapture (SECR) for the leopard camera trapping data

| Region | Parameter | Posterior Mean | Posterior SD | 95% Lower HPD | 95% Upper HPD |
| --- | --- | --- | --- | --- | --- |
| Western Shore | density | 8.420 | 1.030 | 6.770 | 10.420 |
|  | sigma | 1942.895 | 190.199 | 1585.026 | 2328.820 |
|  | lam0 | 0.030 | 0.006 | 0.018 | 0.043 |
|  | beta | 0.000 | 0.000 | 0.000 | 0.000 |
|  | psi | 0.334 | 0.061 | 0.217 | 0.454 |
|  | N | 32.336 | 3.946 | 26.000 | 40.000 |
| Eastern Shore | density | 7.40 | 0.80 | 6.10 | 9.10 |
|  | sigma | 2078.585 | 220.037 | 1655.006 | 2513.231 |
|  | lam0 | 0.030 | 0.007 | 0.018 | 0.044 |
|  | beta | 0.000 | 0.000 | 0.000 | 0.000 |
|  | psi | 0.302 | 0.064 | 0.179 | 0.428 |
|  | N | 19.466 | 2.211 | 16.000 | 24.000 |
| Tembe | density | 4.80 | 0.50 | 3.80 | 5.70 |
|  | sigma | 2428.962 | 199.977 | 2070.303 | 2853.059 |
|  | lam0 | 0.026 | 0.004 | 0.018 | 0.033 |
|  | beta | 0.000 | 0.000 | 0.000 | 0.000 |
|  | psi | 0.222 | 0.041 | 0.142 | 0.302 |
|  | N | 32.698 | 3.621 | 26.000 | 39.000 |
| Ndumo | density | 1.610 | 0.620 | 1.260 | 2.930 |
|  | sigma | 4949.270 | 2681.810 | 1830.860 | 13379.11 |
|  | lam0 | 0.003 | 0.001 | 0.001 | 0.006 |
|  | beta | 0.000 | 0.000 | 0.000 | 0.000 |
|  | psi | 0.195 | 0.096 | 0.043 | 0.379 |
|  | N | 3.855 | 1.472 | 3.000 | 7.000 |

Density = number of individuals per 100 km^2^, sigma = Spatial scale parameter, lam0 = lambda expected encounter frequency (Bayesian) at trap location considered as home range centre, Psi = site occupancy, N = Population size of individuals having their activity centres within the effective trapping area with 5 km buffer.

**Table S3.** A priori models (GLM, Negative Binomial family) for measuring the influence of covariates on estimates of leopard abundance

| Model | K | LogLik | AICc | **∆** AIC | AIC wt |
| --- | --- | --- | --- | --- | --- |
| Poaching | 2 | -45.29 | 94.63 | 0.00 | 0.23 |
| DFT+Poaching | 3 | -44.79 | 95.71 | 1.07 | 0.14 |
| Lion+Poaching | 3 | -44.97 | 96.07 | 1.43 | 0.11 |
| Mediumprey+Poaching | 3 | -45.20 | 96.53 | 1.90 | 0.09 |
| DFT+Lion+Poaching | 4 | -44.59 | 97.39 | 2.76 | 0.06 |
| DFT+Mediumprey+Poaching | 4 | -44.76 | 97.72 | 3.09 | 0.05 |
| Hyaena+Lion+Poaching | 4 | -44.88 | 97.97 | 3.34 | 0.04 |
| CLF+Lion+Poaching | 4 | -44.97 | 98.14 | 3.51 | 0.04 |
| Hyaena+Mediumprey+Poaching | 4 | -45.05 | 98.31 | 3.68 | 0.04 |
| DFT+G+Hyaena | 4 | -45.17 | 98.55 | 3.92 | 0.03 |
| DFT+Hyaena+Lion | 4 | -45.34 | 98.90 | 4.26 | 0.03 |
| DFT+Lion+Poaching | 4 | -45.51 | 99.23 | 4.60 | 0.02 |
| DFT+Hyaena+Lion+Poaching | 5 | -44.47 | 99.25 | 4.62 | 0.02 |
| DFT+Hyaena+Poaching+Smallprey | 5 | -44.60 | 99.50 | 4.87 | 0.02 |
| DFT+Largeprey+P+Poaching | 5 | -44.62 | 99.55 | 4.92 | 0.02 |
| Largeprey+Lion+Mediumprey+Poaching | 5 | -44.84 | 99.98 | 5.35 | 0.02 |
| Lion+MCT+P+Poaching+SF | 6 | -44.58 | 101.61 | 6.97 | 0.01 |
| Lion+MCT+P+Poaching+W | 6 | -44.81 | 102.06 | 7.43 | 0.01 |
| G+Hyaena+MCT+Poaching+Smallprey | 6 | -45.02 | 102.49 | 7.86 | 0.00 |
| DFT+G+Largeprey+Lion+Smallprey | 6 | -45.19 | 102.81 | 8.18 | 0.00 |
| Hyaena+largeprey+P+Poaching+SF+W | 7 | -44.55 | 103.69 | 9.06 | 0.00 |
| G+Hyaena+Largeprey+MCT+Poaching+W | 7 | -45.04 | 104.67 | 10.03 | 0.00 |
| CLF+DFT+largeprey+P+Path+Poaching+SF | 8 | -44.37 | 105.50 | 10.87 | 0.00 |
| CLF+DFT+G+Lion+Path+SF+Smallprey | 8 | -45.01 | 106.77 | 12.14 | 0.00 |
| CLF+DFT+Hyaena+Lion+Mediumprey+P+Poaching+Smallprey | 9 | -44.35 | 107.65 | 13.01 | 0.00 |
| CLF+DFT+G+Lion+MCT+Mediumprey+Path+W | 9 | -44.79 | 108.54 | 13.91 | 0.00 |
| DFT+G+Hyaena+Lion+MCT+Mediumprey+Poaching+SF+W | 10 | -44.25 | 109.68 | 15.05 | 0.00 |
| CLF+G+largeprey+MCT+Mediumprey+P+Path+Poaching+W | 10 | -44.78 | 110.73 | 16.09 | 0.00 |
| CLF+DFT+G+Lion+MCT+Path+Poaching+SF+Smallprey+W | 11 | -44.26 | 111.93 | 17.30 | 0.00 |
| CLF+DFT+G+Hyaena+Largeprey+MCT+Mediumprey+Path+Poaching+Smallprey+W | 12 | -44.34 | 114.36 | 19.72 | 0.00 |
| CLF+DFT+G+Hyaena+Largeprey+Lion+MCT+Mediumprey+P+Path+Poaching+SF+Smallprey+W | 15 | -44.07 | 120.78 | 26.15 | 0.00 |

AIC = Rankings are based on Akaike’s Information Criterion, K = Number of parameters in the model, ∆AIC: Delta Akaike Information Criterion, LogLik = Log likelihood, AIC wt = AIC model weight, AICc = Adjusted AIC for small sample size, CLF= Coastal lowland forest, DFT= Dry forest thicket, MCT= Makatini clay thicket, DF = Dune forest, G = Grassland, P = Plantation, SF = Sand forest and W = Woodland
